# Supplementary material for: A Global Screen for Assembly State Changes of the Mitotic Proteome by SEC-SWATH-MS
Source: Cell Syst. 2020 Feb 26;10(2):133–155.e6. doi: 10.1016/j.cels.2020.01.001 (PMC7042714; doi:10.1016/j.cels.2020.01.001)

Q9H9B1 | EHMT1_HUMAN | EHMT1 EUHMTASE1 GLP KIAA1876 KMT1D
Monomer MW [kDa]: 141.466 Monomer expected elution fraction: 37

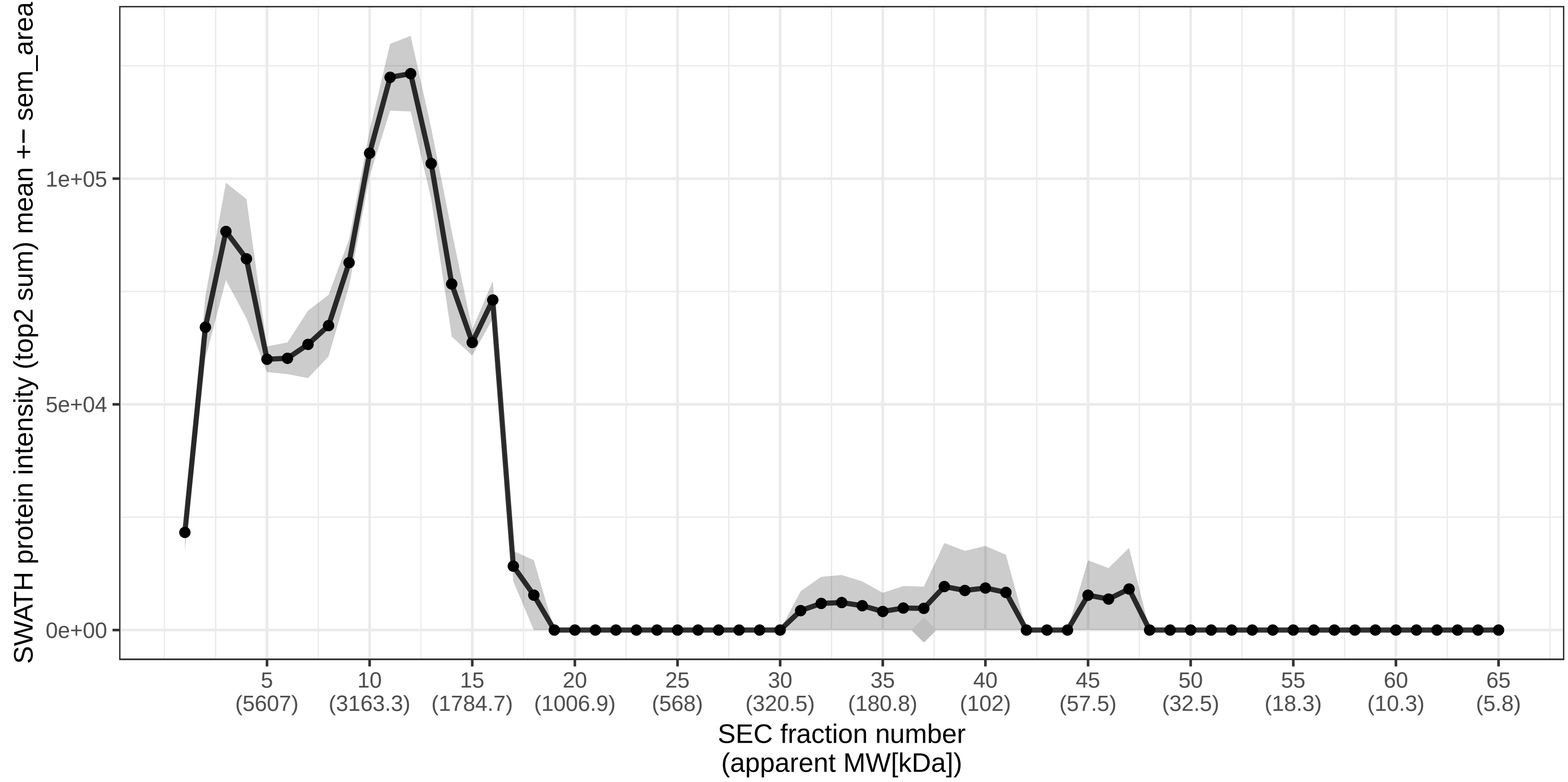

Supplement: Data S1. SEC-SWATH-MS Protein Chromatograms, Related to Figure 1 [file mmc6.zip › SECchrom_Q9H9B1_EHMT1_HUMAN_EHMT1_EUHMTASE1_GLP_KIAA1876_KM.pdf]
